# Parenting and Children’s Behavior During the COVID 19 Pandemic: Mother’s Perspective

**DOI:** 10.3389/fpsyg.2022.801614

**Published:** 2022-04-11

**Authors:** Jael Vargas Rubilar, María Cristina Richaud, Viviana Noemí Lemos, Cinthia Balabanian

**Affiliations:** ^1^National Council for Scientific and Technical Research (CONICET), Buenos Aires, Argentina; ^2^Centro Interdisciplinario de Investigaciones en Ciencias de la Salud y del Comportamiento (CIICSAC), Universidad Adventista del Plata, Libertador San Martín, Argentina

**Keywords:** parenting, children’s behavior, mothers, pandemic, telework, stress

## Abstract

Since the onset of the COVID-19 pandemic, many parents have felt anxious, overwhelmed, and stressed out due to the changes in education and family and working routines. This work aimed to (a) describe three dimensions of perceived parenting (positive parenting, parenting stress, and parental school support) in the COVID-19 pandemic context, (b) describe possible changes perceived by mothers in their children’s behavior during the social isolation phase, (c) analyze if behavioral changes vary according to the dimension of perceived parenting, and (d) analyze whether the characteristics of perceived parenting dimensions vary with mother’s age, number of children and number of work hours. The purposive sample consisted of 646 mothers of school-aged children in Argentina. Questionnaires on sociodemographic and work-related data, and on children’s behavior were administered, as well as an instrument (Vargas Rubilar et al., 2021) that assessed the three parenting dimensions (positive parenting, parenting stress, and parent-school support). The sociodemographic and work-related variables of the study were described using descriptive statistics: measures of central tendency, frequencies, and percentages. The changes perceived in children’s behavior according to the reports given by the mothers regarding positive parenting, parenting stress, and school support were compared using the Mann Whitney’s *U* test, respecting the qualitative nature of the evaluated indicators. A factorial MANOVA was conducted to analyze the effect of mother’s age, ä number of children, and the number of work hours on parenting perceived by mothers. Parenting dimensions influenced the perceived children’s behavior. Mothers with higher positive parenting perceived more changes in their children’s behavior. In addition, those mothers who were more stressed out perceived more problems in almost all the measured behaviors than less stressed mothers. The mothers who reported to have provided more school support to their children perceived that they adapted better to online classes. Finally, mothers’ age and the number of children I parenting, particularly on parenting stress and school support, whereas work hours did not. A number of children affected stress and school support, and age only affected parenting stress. The only significant interaction regarding parenting was observed between the number of children and the number of work hours, which specifically affected parenting stress. Although social isolation due to COVID-19 affected children’s behavior, according to mothers, this might be partially linked to the number of children, mothers’ age, and the mothers’ parenting style. These initial findings may allow the identification of some protective factors and some risk factors of parenting in the Argentine context of a pandemic, and the design of preventive psychoeducational interventions to optimize the psychological wellbeing of families.

## Introduction

The coronavirus disease (COVID-19) pandemic has rapidly extended throughout the world and continues to affect people’s physical and mental health. On March 11, 2020, the World Health Organization declared the COVID-19 outbreak a global pandemic. A few days later, the national government of Argentina ordered compulsory social isolation to prevent contagion and mitigate the virus spread, while strengthening the health system; social isolation was prolonged for several months (from late March to December 2020). However, the healthcare workers knew that trained human resources were not enough and that it was not possible to train them in such a short period. All of this, combined with a very fragile economic situation, aggravated by social isolation that could hardly be implemented by people who needed to go out to work, the added precarious living conditions in a large part of the population (Ernst and López Mourelo, 2020), concentrated in Buenos Aires city and its surroundings (Metropolitan Area of Buenos Aires), where the coronavirus did not take long to spread, led the healthcare workers to believe the numbers of infections would increase significantly and the health system could collapse (Richaud et al., 2021).

During that period, there was no antivirus treatment or vaccines available in Argentina; therefore, isolation and social distancing measures were essential to mitigate the sanitary impact of COVID-19. There was general closure of schools, and classes were given online. Because of these conditions, several teachers and professors reported stress and burnout indicators (Vargas Rubilar and Oros, 2021). Displacement was allowed only for essential workers (i.e., health personnel, security forces, personnel of wholesalers, and retailer food shops, among others). This lockdown lasted approximately eight months, causing an emotional (Canet-Juric et al., 2020) and economic impact on the general population (Ernst and López Mourelo, 2020).

Regarding parenting, for many months an important number of parents felt anxious, overloaded, and stressed out (Almeida et al., 2020; Brown et al., 2020; Olhaberry et al., 2021; Roos et al., 2021), partly due to the changes involved in online education, and in the new family and working routines (e.g., home working) (Almeida et al., 2020; Cluver et al., 2020; UNICEF, 2020). In this sense, a few recent studies (e.g., Brown et al., 2020; Griffith, 2020; Roos et al., 2021) have warned about psychosocial changes generated by the COVID-19 pandemic and their possible mid-and long-term negative impact on children and families. Therefore, becoming aware of the impact of stress associated with the pandemic helps to better understand how the external and internal stressors of families may increase the risk of negative parenting practices for children’s development. In this line, some studies show that high levels of cumulative stress favor rigid and abusive parenting behaviors (Yang, 2015; Hutchison et al., 2016; Liu and Merritt, 2018). In the current context, the closure of schools and the social isolation measures due to the pandemic predisposed to higher parenting stress and parental burnout, which indirectly turned into a potential risk factor for child maltreatment (Griffith, 2020; Ramaswamy and Seshadri, 2020; Lee et al., 2021).

A research conducted in the United States of America (Pew Research Center, 2020) indicates that, of those parents who continue working, over a third (35%) report to be “struggling” to handle the responsibilities of caring for children adequately. Many have had to deal with a new balance between full-time parenting and online education while simultaneously working from their homes (i.e., working from home). The same is true for those parents who perform essential tasks in their workplaces (e.g., hospitals, clinics, supermarkets, and pharmacies), which has exposed them to high levels of risk. All these factors likely contribute to high levels of intrafamily stress. Socioeconomic problems derived from the pandemic also increased the feelings of fear and uncertainty in parents (Pew Research Center, 2020).

Education was also negatively affected by school closure, as it happened in other similar critical public health situations (Braunack-Mayer et al., 2013) or natural disasters (Shavers, 2005). For example, during the outbreak of severe acute respiratory syndrome (SARS) and influenza A (H1N1), some studies reported that parents had work problems and children had school problems, as well as difficulties in communication between school and parents (O’Sullivan et al., 2009; Boon et al., 2011; Braunack-Mayer et al., 2013). In particular, during the COVID-19 pandemic, the lack of face-to-face school activities interrupted the direct contact of children with other adults, such as teachers, counselors, and social workers (Sacks and Jones, 2020). For this reason, the role of parents in terms of school support has been essential, especially for elementary school students, who require more supervision and support to study and do schoolwork. The positive effects of school support by parents have been indicated (Perkins et al., 2016), particularly during the COVID-19 pandemic (e.g., Klootwijk et al., 2021; Lee et al., 2021).

There are records in the literature (e.g., Sprang and Silman, 2013) indicating that social isolation measures during pandemics or natural disasters may be traumatic both for parents and children. Specifically, 30% of children and 25% of parents were found to meet the criteria of post-traumatic stress disorder (e.g., Sprang and Silman, 2013). In this line, a recent longitudinal study (Westrupp et al., 2021) involving Australian parents showed significantly worse mental health in family members due to the consequences of the COVID-19 pandemic. In comparison with the pre-pandemic estimates, fathers had higher rates of symptoms of mental health problems, higher parenting irritability, lower positive family expressiveness, and higher alcohol consumption during the pandemic.

The pandemic seems to have had a more negative impact on maternal parenting (UNICEF, 2020). Mothers have suffered from a greater load of unpaid work and the demand of multitasking concerning their jobs, housework and children care, especially in Latin American cultures (Almeida et al., 2020).

Additionally, there is evidence (Wang et al., 2020) showing that the changes induced by isolation might have a negative impact on children’s physical and mental health, due to the lack of outdoor activities, frustration, and boredom (Olhaberry et al., 2021). In this line, a study that analyzed the impact of quarantine on Italian and Spanish children and adolescents (Orgilés et al., 2020) reported that 85.7% of parents perceived changes in their children’s emotional state and behavior during quarantine. The most frequent symptoms were difficult to focus (76.6%), boredom (52%), irritability (39%), restlessness (38.8%), nervousness (38%), feeling of loneliness (31–3%), uneasiness (30.4%) and worries (30.1%). Children of both nationalities used screens more frequently spent less time doing physical activity and slept more hours during quarantine. Moreover, family coexistence during quarantine was perceived as more difficult. When the pandemic situation became more severe (i.e., a higher number of COVID-19 cases and deaths), the level of stress was higher and parents tended to report more emotional problems in their children (Wang et al., 2020).

On the other hand, type of parenting may favor or negatively affect the children’s response to a stressful event (Bornstein, 2007; Sanders and Turner, 2018). Indeed, positive parenting is characterized by parental practices that promote healthy family relationships and optimize the potential development and wellbeing of children, even under adverse situations (Bornstein, 2007; Rodrigo, 2010; Vargas-Rubilar et al., 2020). From this perspective, positive parenting might be considered an important protective and facilitating factor of parent and family resilience (Walsh, 2004; Miller-Graff et al., 2020) in the pandemic context. In this line, Yamaoka et al. (2021) found that positive parental behaviors, especially those showing empathy, were associated with a lower risk of abusive behavior during the pandemic. By contrast, abusive parent behaviors were associated with greater use of screens, mental health problems of mothers, and intrafamily violence. However, other studies indicated the reciprocal nature of interaction processes between parents and children, i.e., parents may be influenced by children’s attitudes and behaviors as well (Biglan, 2015). For example, children that are more irritable, or have sleep or behavior problems usually elicit more negative responses from their parents (McQuillan and Bates, 2017).

Furthermore, some sociodemographic and work-related characteristics of parents might affect parenting. For instance, a study involving Spanish parents showed that being the father or mother of multiple children favors the perception of higher parenting stress (Pérez et al., 2010). Likewise, although a high number of hours dedicated to work may be viewed as negative to parenting, Hughes and Parkes (2007) showed that this relation is mediated by different protective factors, such as social support. Working longer hours could interfere with family satisfaction only when employees have little or no control over their working schedule (Hughes and Parkes, 2007).

Regarding the effect of age on parenting, some studies about parenting styles in young mothers (i.e., under age 21 at childbirth) suggest that many are not sufficiently prepared to provide sensitive and positive parenting (Easterbrooks et al., 2011). Another study points out that parents between 30 and 40 years of age are more likely to adequately satisfy the needs of their children (e.g., Bezeveggis, 2012).

Overall, most of the studies about parenting conducted in different countries during the pandemic have focused on the parenting stress and burnout generated by this phenomenon (e.g., Azhari et al., 2020; Brown et al., 2020; Griffith, 2020; Westrupp et al., 2021). Other works analyzed the mental health problems of parents and children (e.g., Almeida et al., 2020; Wang et al., 2020; Escobar et al., 2021; Roos et al., 2021; Westrupp et al., 2021). Some reports addressed changes in children’s behavior (Jiao et al., 2020; Orgilés et al., 2020; Oliveira et al., 2021) or the role of parenting in online education (e.g., Klootwijk et al., 2021; Lee et al., 2021).

However, few works have addressed the potential protective role of positive parenting practices (e.g., expression of affection, communication, and school support) in such an adverse context. To our knowledge, there are no works, especially for Argentina, that analyze the perceived parenting in mothers of schoolchildren considering the education conditions in the pandemic context. Finally, to date, no studies dealing with both the family characteristics as protective factors (i.e., positive parenting and school support) and risk factors (parenting stress, specific work, and sociodemographic characteristics) have been conducted in the pandemic context in Argentina.

Therefore, this study aims to: (a) describe three dimensions of perceived parenting (positive parenting, parenting stress, and parental school support) in the context of the COVID-19 pandemic, (b) describe possible changes perceived by mothers in their children’s behavior during the phase of social isolation, (c) analyze if changes in behavior vary as a function of the perceived parenting dimensions, and (d) analyze whether the three dimensions of perceived parenting vary according to mother’s age, number of children and the number of work hours.

For the latter two aims, which require inferential analyses, we formulate the following hypotheses:

Hypothesis 1: Changes in children’s behavior varies depending on the parenting perceived by the mother.

Hypothesis 2: Perceived parenting varies depending on the age of the mothers, the number of children, and the number of hours of work.

## Materials and Methods

### Type of Study and Design

The conducted study was quantitative, descriptive, and cross-sectional (Bickman and Rog, 1998).

### Participants

Sampling was conducted using a non-probabilistic availability sampling method (Otzen and Manterola, 2017). The final sample consisted of 646 mothers, aged between 22 and 59 years (*M* = 37.62; *SD* = 5.50) from different regions of Argentina. Inclusion criteria were that mothers were over 18 years of age and that had school-age children (between 5 and 12 years old). Mothers of children with psychological, developmental, or learning disorders were not included in the study.

Of the whole sample, 84% of the mothers were married or had a partner, 9% were separated or divorced, and 7% were single. Concerning educational level, 48% had university studies, 27% had tertiary studies, 15% had high school studies, and 3% mentioned not having primary education or academic training. Lastly, 7% were pursuing or had completed postgraduate studies (see Table 1).

**TABLE 1:** Demographic and work-related characteristics of the participants.

		*N*	*%*
Civil status	Married	381	59.0
	In consensual union	162	25.0
	Separated/Divorced	53	8.2
	Single	44	6.8
	Other	6	1.0
Occupation	Homemaker	123	19.0
	Unemployed	23	3.6
	Employed	148	22.9
	Self-employed	352	54.5
Number of work hours	Less than 6 h	257	39.8
	Between 6 and 10 h	272	42.1
	More than 10 h	117	18.1
Academic studies	No academic studies	2	0.3
	Primary school	14	2.2
	Secondary school	97	15.0
	Tertiary education (non-university level)	178	27.5
	University degree	309	47.8
	Postgraduate studies (incomplete)	23	3.6
	Postgraduate studies (complete)	23	3.6
Number of children	1	148	22.9
	2	350	54.2
	3	118	18.3
	4 or more	30	4.6

### Instruments

Questionnaire about sociodemographic and work-related characteristics. Data on the sociodemographic and work-related characteristics of the participants were collected using an *ad hoc* semi-structured questionnaire that included questions about age, civil status, educational level, number of children, and number of work hours.

Questionnaire about children behavior as perceived by mothers. To know children’s behavior as perceived by mothers during the quarantine due to COVID-19, an *ad hoc* structured questionnaire was created. Firstly, mothers were asked whether they had observed changes in their children’s behavior. The answer options were “yes” or “no.” The following question was: Have you observed that those changes in the behavior of your children were…? The answer options were: “less,” “the same as” or “more” than before (e.g., *He/She sleeps less than before, He/She sleeps the same as before, He/She sleeps more than before*). As detailed in Table 2, the analyzed behaviors included: (a) children’s behaviors observed in the family environment to sleep, eating habits, mood, relationship with peers, and (b) children’s behaviors observed in the current school situation: online classes, relationship with classmates, compliance with school assignments.

**TABLE 2:** Frequency of behaviors behavior compared to pre-pandemic times and percentage of change in children’s behavior.

	Less than before		The same as before		More than before	
	*n*	*%*	*n*	*%*	*n*	*%*
Sleeps	121	18.7	283	43.8	242	37.5
Is sad	89	13.8	244	37.8	313	48.5
Eats	60	9.3	321	49.7	265	41.0
Disobeys	66	10.2	230	35.6	350	54.2
Fights with siblings	77	11.9	292	45.2	277	42.9
Is anxious/nervous	54	8.4	167	25.9	425	65.8
Screams	67	10.4	236	36.5	343	53.1
Wants to sleep in my/our bed	105	16.3	280	43.3	261	40.4
Shows dependent behavior	82	12.7	267	41.3	297	46.0
Shows defiant behavior	68	10.5	201	31.1	377	58.4
Plays with friends (now online)	265	41.0	179	27.7	202	31.3
Once asleep, he/she wakes up confused in the middle of the night	222	34.4	320	49.5	104	16.1
Has nightmares	226	35.0	304	47.1	116	18.0

The dimensions of perceived parenting during the pandemic were evaluated using the *Brief scale of perceived parenting during the pandemic* provided by Vargas Rubilar et al. (2021). The instrument operationalizes three dimensions of perceived parenting in the pandemic context: (a) positive parenting (e.g., *I have managed to maintain a family atmosphere that is good for the development of my children, despite everything that has happened*), (b) parenting stress (e.g., *Keeping an eye on my children’s classes and assignments stress me out*.), and (c) parental school support (e.g., I *keep frequent contact-communicate with school during online education so I am up to date on how my children are doing*), through 17 statements (see Table 1) that were answered utilizing a 4-point Likert scale (*Never, Seldom, Very often, Always*), The previous study of the instrument involving Argentine mothers indicated suitable psychometric properties (Vargas Rubilar et al., 2021). The confirmatory study of factorial structure of three factors of the scale showed satisfactory fit indexes (χ^2^/dg = 1.22; NFI = 0.93; NNFI = 0.99; CFI = 0.99; IFI = 0.99; GFI = 0.99) and an acceptable error (RMSEA = 0.02). Similarly, internal consistency was adequate for the three dimensions: positive parenting (ω = 0.79), parenting stress (ω = 0.77), and parental school support (ω = 0.75) (Vargas Rubilar et al., 2021).

### Ethical Procedures and Data Collection

Mothers participated anonymously and gave their consent before answering the questionnaire. The collected information was handled with confidentiality and no access was given to people outside the research. The actions performed in the setting of this study complied with the international ethic recommendations for research with human subjects (American Psychological Association, 2017). To prevent survey fatigue, a reduced number of questions was used, estimating a span of no longer than 10 min for participants to answer.

Data were collected between September and December 2020, during the quarantine phase, 5 months after compulsory social isolation was mandated in Argentina. During this period, all the schools were closed and classes were given only online. Due to the particular conditions of social isolation under which the study was conducted, mothers of primary school children were invited to participate through social networks (Facebook, Instagram, etc.), e-mail (Gmail, Outlook, etc.), and chat groups of mothers of school grades in digital messaging services (e.g., WhatsApp, Telegram, etc.). The mothers that voluntarily agreed to participate signed the informed consent and then received a link to the questionnaire. The evaluation was made online through a form (*Google Forms*) that included the described instruments.

### Procedures for Data Analysis

The sociodemographic and work-related variables of the study were analyzed using descriptive statistics: measures of central tendency (mean, standard deviation), frequencies, and percentages.

The changes perceived in the behavior of children as reported by the mothers in terms of positive parenting, parenting stress, and parental school support were compared using the Mann Whitney’s *U* test, respecting the qualitative nature of the evaluated indicators.

For this analysis, each parenting dimension was previously categorized into three groups (i.e., high, medium, and low, with two cut-off points at the 33 and 66 percentiles, following the criterion of homogeneous frequencies per group). Then the high and low groups of each dimension (i.e., positive parenting, parental school support, and parenting stress) were selected to analyze the perceived children’s behavior relative to those dimensions.

A factorial MANOVA was conducted to analyze the effect of age, the number of children, and the number of work hours in the parenting perceived by mothers.

Data were analyzed using the software *SPSS* version 25.

## Results

### Description of Parenting Perceived by Mothers

Mean and standard deviation values of each dimension and indicator that was included to assess parenting perceived by mothers are presented in Table 3. The item that obtained the highest value was *I genuinely express my love to my children*, of the positive parenting dimension. The item that presented the lowest rating was *The main source of stress in my life is my children*, of parental stress dimension.

**TABLE 3:** Scores of the Perceived Parenting Scale items and dimensions (expressed as Mean and Standard Deviation).

	*M*	*SD*
***Positive parenting***	3.40	0.42
I try that each member of the family expresses their opinions and/or encourages them to do. so	3.47	0.62
I genuinely express my love to my children.	3.68	0.54
I talk with my children about their mistakes.	3.63	0.57
I help my children build a daily hygiene routine.	3.40	0.66
I have managed to maintain a family atmosphere that is good for the development of my children, despite everything that has happened.	3.08	0.69
I dedicate some time during the day to speak to my children.	3.17	0.71
I take time to meet my children’s needs.	3.46	0.63
I stimulate my children to do recreational and artistic activities away from the screens.	3.30	0.71
***Parenting stress***	*2.56*	*0.64*
Keeping an eye on my children’s classes and assignments stresses me out.	3.12	0.86
The main source of stress in my life is my children.	2.14	0.89
I don’t have enough time, as I used to do, to fulfill all my responsibilities.	2.79	0.95
Because of my children, I find it difficult to balance different responsibilities.	2.39	0.94
I have had trouble sleeping during the pandemic.	2.33	0.98
I am more irritable during the pandemic.	2.57	0.90
***Parental school support***	*3.35*	*0.64*
I know which homework and assignments are given to my children in online education.	3.51	0.72
I keep in frequent contact- communicate with school during online education, so I am up to date on how my children are doing.	3.28	0.84
I help get my children organized regarding daily study time.	3.27	0.77

*M, mean; SD, standard deviation.*

### Description of the Behavior Observed in Children

The different behaviors that mothers observed in their children are presented in Table 2, with their corresponding frequency (less than, the same as or more than before) of observation during the period of mandatory isolation enacted due to the COVID-19 pandemic. The children’s behaviors that had the greatest increase during the isolation, as perceived by more than 50% of mothers, were *Is anxious/nervous, Shows defiant behavior, Disobeys.*

### The Behavior of Children According to Perceived Parenting

The analysis of Tables 4–6 shows the variation in children’s behaviors according to the level of positive parenting, parenting stress, and parental school support informed by mothers, respectively.

**TABLE 4:** Change in children’s behavior according to the level of positive parenting.

Variable	Low parenting		High parenting		Statistics	
	Mean range	Range addition	Mean range	Range addition	*U*	*p*
Sleeps	214.66	53021.50	220.10	40939.50	22393.50	0.631
Is sad	224.87	55543.50	206.54	38417.50	21026.50	0.109
Eats	214.53	52990.50	220.27	40970.50	22362.50	0.606
Disobeys	229.06	56577.00	200.99	37384.00	19993.00	0.015
Fights with siblings	231.17	57099.50	198.18	36861.50	19470.50	0.004
Is anxious/nervous	230.01	56812.50	199.72	37148.50	19757.50	0.008
Screams	236.39	58388.50	191.25	35572.50	18181.50	0.000
Wants to sleep in my/our bed	221.02	54591.50	211.66	39369.50	21978.50	0.417
Shows dependent behavior	229.27	56630.00	200.70	37331.00	19940.00	0.013
Shows defiant behavior	228.53	56446.00	201.69	37515.00	20124.00	0.021
Plays with friends (now online)	213.99	52855.00	221.00	41106.00	22227.00	0.554
Once asleep, he/she wakes up confused in the middle of the night	212.05	52376.50	223.57	41584.50	21748.50	0.304
Has nightmares	214.36	52946.50	220.51	41014.50	22318.50	0.588

**TABLE 5:** Change in children’s behavior according to the level of parenting stress.

Variable	Low stress		High stress		Statistics	
	Mean range	Range addition	Mean range	Range addition	*U*	*p*
Sleeps	225.71	46721.50	229.00	56563.50	25193.50	0.778
Is sad	186.94	38696.50	261.49	64588.50	17168.50	0.000
Eats	220.39	45621.00	233.46	57664.00	24093.00	0.251
Disobeys	184.14	38116.50	263.84	65168.50	16588.50	0.000
Fights with siblings	192.42	39831.00	256.90	63454.00	18303.00	0.000
Is anxious/nervous	178.50	36949.50	268.56	66335.50	15421.50	0.000
Screams	178.49	36947.00	268.57	66338.00	15419.00	0.000
Wants to sleep in my/our bed	196.54	40684.00	253.45	62601.00	19156.00	0.000
Shows dependent behavior	193.44	40043.00	256.04	63242.00	18515.00	0.000
Shows defiant behavior	176.43	36520.50	270.30	66764.50	14992.50	0.000
Plays with friends (now online)	234.99	48643.50	221.22	54641.50	24013.50	0.253
Once asleep, he/she wakes up confused in the middle of the night	207.01	42851.50	244.67	60433.50	21323.50	0.001
Has nightmares	206.18	42679.50	245.37	60605.50	21151.50	0.001

**TABLE 6:** Change in children’s behavior according to the level of parental school support.

Variable	Low support		High support		Statistics	
	Mean range	Range addition	Mean range	Range addition	*U*	*p*
Logs in to take online classes	174.11	39870.50	259.29	51080.50	13535.50	0.000
Does the homework	161.72	37033.00	273.70	53918.00	10698.00	0.000
Joins classmates online to do the schoolwork and/or study	209.40	47953.50	217.20	42571.50	21618.50	0.467
Enjoys online classes	185.29	42432.50	246.29	48518.50	16097.50	0.000
Gets easily frustrated when doing school assignments	228.09	52232.00	196.54	38719.00	19216.00	0.006

As can be observed in Table 4, the behaviors in which that differed between high and low positive parenting groups were: *He/She is disobedient, He/She fights with siblings, He/She is anxious, He/She screams, He/She shows dependent behavior, He/She shows defiant behavior*. For all these behaviors, the mean range was higher in mothers who perceived low positive parenting.

Differences observed in the behavior of children between high and low parenting stress groups were detected in the following behaviors: *He/She is sad, He/She disobeys, He/She fights with siblings, He/She is anxious/nervous, He/She screams, He/She wants to sleep in my/our bed, He/She shows dependent behavior, He/She shows defiant behavior, Once he/she falls asleep, he/she wakes up confused in the middle of the night*, and *He/She has nightmares*. In all cases, as can be observed in Table 5, the mean range was higher in mothers who perceived a high level of parenting stress.

Lastly, the behavior of children showing differences regarding the level of parental school support was: *He/She logs in to take online classes, He/She does the homework, He/She enjoys online classes, He/She gets easily frustrated when completing school assignments*. In this case, the positive behaviors were linked to high levels of parental support, and the behavior of frustration regarding the compliance with school assignments was more often observed in mothers with lower parental school support (see Table 6).

### Influence of Sociodemographic and Work-Related Variables on Perceived Parenting

The effect of the mothers’ age, the number of children, and the number of work hours (i.e., independent variables) on the parenting (i.e., positive parenting, parenting stress, and parental support school: dependent variables) perceived by mothers was analyzed using a factorial MANOVA. The results indicate that the number of children had an impact on parenting [*Hotelling’s F*_(6,1232)_ = 4.63; *p* < 0.001]. Mothers’ age also had a significant effect [*Hotelling’s F_(6,1232)_* = 4.24; *p* < 0.001]. However, the number of work hours did not have a significant effect on parenting [*Hotelling’s F*_(6,1232)_ = 0.62; *p* = 0.714].

The univariate analyses show that the number of children specifically affects parenting stress [*F*_(2,619)_ = 4.99; *p* = 0.007]. Mothers who had 3 or more children presented higher values of the parental stress dimension than those with 1 or 2 children. The school support dimension also showed differences [*F*_(2,619)_ = 8.55; *p* < 0.001]. Mothers with 2 children had higher values in the school support dimension than mothers with 3 or more children. Mother’s age only affected parenting stress [*F*_(2,619)_ = 7.25; *p* = 0.001]. Indeed, younger mothers (between 22 and 34 years old) had higher values of parenting stress dimensions than 35 to 45 year-old mothers and between 46 and 59-year-old mothers (see Tables 7, 8).

**TABLE 7:** Effect of number of children on perceived parenting.

	Number of children						*F*	*ETA*	*M*_1_-*M*_2_	*M*_1_-*M*_3_	*M*_2_-*M*_3_

	1 child		2 children		3 or more						
	*M* ^1^	*SD* ^1^	*M* ^2^	*SD* ^2^	*M* ^3^	*SD* ^3^					
Positive parenting	3.41	0.05	3.41	0.03	3.30	0.04	2.35	0.01	0.37	0.02	0.17
Parenting stress	2.49	0.07	2.48	0.05	2.71	0.06	5.00**	0.02	0.49	0.03	0.16
School support	3.36	0.07	3.46	0.05	3.14	0.06	8.55***	0.03	0.14	0.19	0.00

**p < 0.01 *** p < 0.001 Generalized linear model Hotelling’s F_(6,1232)_ = 4.63; p = 0.00.

**TABLE 8:** Effect of age on perceived parenting.

	Age						*F*	*ETA*	*M*_1_–*M*_2_	*M*_1_–*M*_3_	*M*_2_–*M*_3_

	22–34		35–45		46–59						
	*M* ^1^	*SD* ^1^	*M* ^2^	*SD* ^2^	*M* ^3^	*SD* ^3^					
Positive parenting	3.36	0.04	3.42	0.03	3.34	0.05	1.38	0.00	0.99	0.85	0.78
Parenting stress	2.72	0.06	2.56	0.04	2.39	0.08	7.25***	0.02	0.14	0.00	0.05
School support	3.23	0.06	3.31	0.04	3.42	0.08	1.96	0.01	0.91	0.71	0.86

*** p < 0.001 Generalized linear model Hotelling’s F_(6,1232)_ = 4.24; p = 0.00.

The only significant interaction regarding parenting (after removing non-significant ones from the model) was observed among the factors: number of children by the number of working hours [*Hotelling’s F*_(12,1895)_ = 2.72; *p* = 0.001]. This interaction had specific influence on parenting stress [*F*_(4,635)_ = 3.11; *p* = 0.015]. Differences in parenting stress were found in the group of mothers who worked over 10 h, between mothers who have a single child (*M* = 2.48; *SD* = 0.12) and those who have 3 or more children (*M* = 2.94; *SD* = 0.10) (*p* = 0.014), and between those who have 2 (*M* = 2.43; *SD* = 0.08) and 3 or more children (*p* = 0.001); as expected, there was a higher perception of stress in those mothers who work over 10 h and have 3 or more children.

## Discussion

The COVID-19 pandemic has had a worldwide impact on the population in general, forcing families to restrict physical contact with their loved ones, and affecting the perception of closeness and affection of some family members (Newkirk, 2020). In particular, parents have undergone an increase in parenting stress and vulnerability (Brown et al., 2020; Griffith, 2020). Furthermore, children have been affected by the pandemic at the psychological, social, and family levels (Jiao et al., 2020). Wang et al. (2020) indicate that, during the pandemic, children have done less physical activity and have spent more time using screens; they have had irregular sleeping patterns and less healthy eating habits, while, according to Jiao et al. (2020), they are more irritable and have more difficulty in paying attention and concentrating.

In this context, this study attempted to describe some dimensions of parenting, as well as behavioral changes in children as perceived by mothers in Argentina during the social isolation period. It also attempted to analyze whether certain pre-pandemic characteristics, such as positive parenting, mothers’ age or number of children, as well as work-related characteristics, such as number of work hours during the social isolation (i.e., working from home) or the need to support children to do schoolwork, have been protective or risk factors.

Regarding the first objective (to describe the three dimensions of perceived parenting: positive parenting, parenting stress, and parental school support in the context of the COVID-19 pandemic), the results indicated the predominance of positive parenting. Mothers are characterized by: genuinely expressing their love to their children, speaking to their children about their mistakes, knowing about which homework and assignments are given to their children in online education, try that each member of the family expresses their opinions and/or encourage them to do so, and *take time to meet their children’s needs*. These behaviors summarize an authoritative parenting style (Baumrind, 1966) or a positive parenting style (Bornstein, 2007), implying high acceptance and moderate control. In general, these mothers showed fewer behaviors denoting stress, although some were more outstanding, such as *keeping an eye on my children’s classes and assignments stress me out, I don’t have enough time, as I used to do*, *to fulfill all my responsibilities*, *I am more irritable during the pandemic*. The latter is in agreement with what was claimed by Almeida et al. (2020) about the overload experienced by mothers during the pandemic, due to the multiple tasks they must perform when trying to balance housework, the care and the school support given to children, and the paid work that is now done from home. By contrast, the behaviors reflecting positive parenting would be based on a more stable pre-pandemic characteristic. At the same time, it should be noted that most of the mothers included in this study have high education levels, and people with this education level would appreciate positive parenting characteristics more than people with lower education (Richaud et al., 2014), which might have influenced our results.

Regarding the second objective (to describe possible changes perceived by mothers in their children’s behavior during the social isolation phase), they usually appear in almost all the studied behaviors, except *He/She has nightmares* and *He/She wakes up confused*. However, sleep is precisely the behavior that showed the greatest differences –either sleeping less or more than usual–, affecting more than half of the children. The most significant differences that were observed by more than half of the mothers were the increase in anxiety and nervousness, followed by the presence of more defiant behavior, more aggressiveness, and difficulties in eating –eating more or less than before the pandemic. These results agree with those of Escobar et al. (2021), who conducted a study involving 5,997 mothers and fathers in the first weeks of quarantine and found that 73.4% of the participants perceived that children were more demanding, as well as an increase in mood-related symptoms (22.8%), disruptive behaviors (49.8%), anxiety symptoms (39.2%), low tolerance to frustration (38%), sleeping problems (52.5%) and symptoms associated with attention deficit and hyperactivity (70.8%). In addition, parents noticed that their children were more defiant (51%) and fought more (32%).

Cabana et al. (2021) conducted work after 8 months of quarantine, involving 4,500 children and adolescents from Argentina. The authors found that almost 77% of children were “angry” and 68% exhibited different degrees of sadness, 7 every 10 children and adolescents (6 to 18 years of age) expressed negative feelings such as low interest and boredom, and 6 every 10 individuals recognized having fear, either for themselves (24%) or for others (21%). Among those that were “annoyed” with the quarantine, the main reason was school work (45%), followed by quarantine-derived measures, especially for the 6-to-9-year-old group (21%). The study found that children felt a high burden in the extraordinary situation they were living and that they were overwhelmed; they perceived a reduction in education quality, in the subject contents; that education was socially unequal and had made use of a “fun and entertainment tool” –online connection through different devices– turning it into part of their duties. Of the activities performed before the quarantine, 60% of the children missed outdoor activities, recreation in general, and sports, especially children between 6 and 9 years old (Cabana et al., 2021).

Although they are “digital natives,” children missed in-person contact with their friends, since digital communication does not replace face-to-face contact. Grandparents appeared as very important actors: they provide support and affection and share special experiences with their grandchildren. These feelings can be easily understood in the Argentine culture, in which face-to-face relationships are highly valued, kissing and hugging among relatives and friends are common, and grandparents are very important in family life (Richaud et al., 2014).

Regarding objective 3, since changes in children’s behavior were reported by mothers, we may hypothesize that their perception might be partially influenced by their perceptions about parenting. For this reason, analyzing whether this perception varied according to the parenting style was an interesting point. When comparing the answers given by mothers with high and low positive parenting, we found differences in their perception of children’s behavior. Mothers with low positive parenting perceived that their children screamed more, fought more with their siblings, were more anxious and disobedient, and showed more defiant and dependent behaviors. This is in agreement with previous findings (Bornstein et al., 1998; Johnston et al., 2018), in that self-assessments (i.e., cognitions, ideas, or beliefs) about parenting of mothers, fathers or caregivers influence their behavior and their children’s beliefs and behaviors. Parents’ thoughts about their children and parenting are an integral aspect of family interactions. Various cognitions of parents, including both stable and general beliefs, expectations, and attributional patterns related to children, child behavior, and parenting, as well as more dynamic cognitions that frequently occur in the context of ongoing parent-child interactions influence his/her perception of his/her children behavior (Johnston et al., 2018).

On the other hand, the comparison between mothers with high and low parenting stress showed that those who were more stressed out perceived more negative changes in the behavior of their children and that those changes usually coincide with those perceived by mothers with low positive parenting, as is the case of disobedience, fighting with siblings, anxiety, and nervousness, screaming and showing defiant behaviors. However, it is worth noting that these more stressed mothers, unlike the other cases, perceive more internalizing behaviors, such as children being sadder, and regressive behaviors (i.e., more dependency, desire to co-sleep or share a bed with parents, having nightmares, and waking up confused). These results agree with those of Olhaberry et al. (2021), who highlight the association between the deterioration in parents’ daily functioning and the perception of deterioration in their children’s daily functioning. This shows the mutual influence among mothers, fathers, and children (Ponnet et al., 2013; Azhari et al., 2020), as well as the tendency to perceive children’s behavior in a more negative way, which stems from their discomfort related to confinement and health restrictions.

However, despite the previous considerations about how the particular mothers’ beliefs, cognitions, feelings, and moods might influence their statements about the change in behavior in children, in general, the reported changes agree with another study conducted in Argentina (Cabana et al., 2021), in which the source of information was the children themselves. Therefore, our results agree with those findings.

Concerning the perception of school support, the mothers who gave more support perceived that their children logged in to online classes more often, completed their assignments, enjoyed their classes, and were less frustrated when doing schoolwork at home. These results agree with previous findings indicating that family support is an important predictor of the children’s bonding and good school performance (Perkins et al., 2016). Likewise, another recent study showed that the lowest levels of parental school support were related to the lower academic motivation of students during the pandemic (Klootwijk et al., 2021).

Based on the results discussed concerning objective 3, it is possible to confirm hypothesis 1: “Changes in children’s behavior varies depending on the parenting perceived by the mother.”

Regarding the last objective, the analysis of the effect of the number of children on positive parenting, parenting stress, and school support showed significant differences between groups of high and low parenting stress, which increases significantly when comparing mothers with 1 and 3 children, and in-school support, which decreases significantly between 2 and 3 children, as previously suggested (Pérez et al., 2010). The number of children might be considered a pre-pandemic stressor. This factor might increase the stress of mothers that have to meet the needs of several children at the same time in a context of greater demand inside the home (e.g., *I don’t have enough time, as I used to do, to fulfill all my responsibilities*). This situation would lead mothers to perceive a higher number of negative behaviors in children; in turn, children perceive greater tension and therefore will exhibit more behavioral problems. As already stated, previous works (Yang, 2015; Hutchison et al., 2016; Liu and Merritt, 2018) found that the accumulation of stressors makes parents’ behavior rigid, which in turn would lead to greater irritability and behavioral problems in children, eliciting more negative responses by their parents (McQuillan and Bates, 2017).

Mothers’ age, had an influence only on parenting stress, decreasing as age increases, especially and significantly between groups 1 (22–34 years old) and 3 (46–59 years old), and between groups 2 (35–45 years old) and 3. These results coincide with those found by Bezeveggis (2012), who indicates that older parents (30–40 years old) are more mature and more likely to meet the needs of their children. Mothers over 30 years old adapt better and are happier in their maternity role (Bezeveggis, 2012) than younger mothers, who have more difficulty in exhibiting positive parenting (Easterbrooks et al., 2011). This situation might also be a pre-pandemic variable that influences maternal stress.

Although the number of work hours did not have a direct impact on parenting, the analysis of the effect of the interaction between work hours and the number of children showed that mothers with three or more children who work more than 10 h a day have higher perceived stress. As previously reported, the relationship between work and family life in women might be mediated by diverse moderating factors, such as flexibility and control of work hours (Hughes and Parkes, 2007). Indeed, a study conducted during the pandemic showed that parents that perceived themselves as more productive reported a higher level of positive emotions (Ilari et al., 2021). In our study, working a high number of hours (more than 10 a day) and having more than three children would seem to increase stress in mothers during the pandemic. Our results agree with previous studies that found that certain socio-demographic factors moderate the psychological impact of quarantine. A particular study (Taylor et al., 2008) found that gender, age, number of children, and educational level were aspects associated with the psychological effect of the quarantine.

Our findings partially confirm hypothesis 2 of this study, since perceived parenting varied depending on mothers’ age and the number of children, but not with the number of work hours.

In summary, during the pandemic, social isolation conditions had a negative effect on mental health and children’s behavior. This impact, however, has been influenced either positively by protective factors, such as positive parenting and school support, or negatively by risk factors, such as younger age and a high number of children, which existed before the pandemic. At the same time, there have been contextual factors, such as lack of social contact or online classes, which have influenced parenting. Therefore, the analysis of the effect of quarantine during COVID 19 on parenting and children’s behavior needs to consider family dynamics, characteristics of parents and parents-children interactions, and sociodemographic and context variables, to be able to determine protective and risk factors that can enhance or reduce the effect.

### Limitations and Strengths

One of the limitations of the work was that it involved self-reports, which may be affected by social desirability. Secondly, a cross-sectional evaluation was conducted which, unlike a longitudinal study, does not allow the accurate detection of changes in the mothers’ parenting and the children’s behavior. Thirdly, the study involved only mothers; possibly, the assessed changes may have different results in mothers with children of other age ranges and social strata. Additionally, only a reduced number of variables have been evaluated, since many relevant families, parental and working characteristics that affect parenting have not been included in this study. Finally, the study of children’s behavior was conducted only from the mother’s perspective. Further studies should analyze and compare the perceptions of both parents as well as of children about parenting and behavior. For this reason, our results cannot be generalized to other samples with different conditions and family characteristics.

On the other hand, the analyses conducted did not consider the following variables, which might have influenced the results: children gender or age, data on parenting before the pandemic, grief due to the loss of a family member during the health emergency, or possible school difficulties that children might have. The effect of children’s behavioral changes on parental stress was also not analyzed, considering that the mother-children interaction is reciprocal or bidirectional (Biglan, 2015). Therefore, future works should include these data.

The main strength of this study lies in the contribution of evidence regarding some dimensions of parenting and the changes in children’s behavior observed by Argentine mothers during the COVID-19 pandemic. In addition, the study has shown a relationship between parenting and the changes perceived by mothers in their children’s behavior, which probably produces negative feedback on the children’s behavior and their parenting. Lastly, the impact of some sociodemographic and work-related factors interacting and having an effect on perceived parenting is highlighted. It is estimated that these initial findings may allow the identification of some protective factors as well as some risk factors to parenting in the context of a pandemic, and the design of preventive psychoeducational interventions to optimize the psychological wellbeing of families.

### Implications

As suggested by Provenzi and Tronick (2020), the evidence obtained from the research should be used to generate strategies promoting psychological reparation and reduce the generated damage, mainly social disconnection during the pandemic. The present results, as well as previous studies (e.g., Roos et al., 2021), evidence the need to generate psychosocial intervention strategies to support parents during and after the pandemic. Specifically, group and individual interventions might be performed to strengthen positive parenting practices, i.e., healthy, protective, and stable emotional relations that provide school support, recreational activities, and high-quality time to children. Moreover, a positive familiar environment, with suitable management of stress and free of verbal, physical, and emotional abuse should be promoted. Intervention programs based on a positive parenting approach have shown encouraging results in adverse contexts and conditions (e.g., Rodrigo, 2010; Pickering and Sanders, 2016; Vargas Rubilar et al., 2018; Turner et al., 2020), and promising results were reported to address management of parenting stress during the COVID-19 pandemic (e.g., James Riegler et al., 2020).

## Conclusion

During the pandemic, the Argentine mothers included in this work perceived adequate positive parenting and moderate stress due to the need to support children learning, as well as to the greater responsibility of having to perform multiple tasks. However, the level of maternal stress was found to depend not only on the pandemic but also on contextual variables, such as the number of children, mother’s age, and the interaction between the number of work hours and the number of children. The mothers that perceived the highest stress were those that perceived more sadness and more dependent behaviors in their children.

## Data Availability Statement

The raw data supporting the conclusions of this article will be made available by the authors, without undue reservation.

## Ethics Statement

The studies involving human participants were reviewed and approved by the Ethics Committee of the Universidad Adventista del Plata. The participants provided their written informed consent to participate in this study.

## Author Contributions

JVR: study design and questionnaire, data collection, introduction, methodology, and discussion. MR: study design, questionnaire, methodology, and discussion. VL: methodology, data analysis, and results. CB: form design, data collection, and results. All authors reviewed the draft and contributed to the final version of the manuscript.

## Conflict of Interest

The authors declare that the research was conducted in the absence of any commercial or financial relationships that could be construed as a potential conflict of interest.

## Publisher’s Note

All claims expressed in this article are solely those of the authors and do not necessarily represent those of their affiliated organizations, or those of the publisher, the editors and the reviewers. Any product that may be evaluated in this article, or claim that may be made by its manufacturer, is not guaranteed or endorsed by the publisher.
